# The clinical significance of preoperative serum fibrinogen levels and platelet counts in patients with gallbladder carcinoma

**DOI:** 10.1186/s12876-021-01943-x

**Published:** 2021-10-07

**Authors:** Peng Cao, Lei Jiang, Liang-Yi Zhou, Yan-Ling Chen

**Affiliations:** 1 The First Affiliated Hospital, Department of Hepatopancreatobiliary Surgery, Hengyang Medical School, University of South China, Hengyang, Hunan, 421001 China; 2 Department of Hepatobiliary Surgery and Fujian Institute of Hepatobiliary Surgery, Fujian Medical University Union Hospital, Fujian Medical University Cancer Center, Fuzhou, China

**Keywords:** Gallbladder carcinoma, Hyperfibrinogenemia, Thrombocytosis, Lymph nodes metastases, Distant metastasis, TNM stage, Prognosis

## Abstract

**Background:**

Gallbladder carcinoma (GBC) was the most common malignancy of biliary tract. Patients with malignancies frequently present with activated coagulation pathways, which might potentially related to tumor progression and prognosis. The purpose of the study was to investigate the clinical significance of preoperative serum fibrinogen levels and platelet counts in GBC patients.

**Methods:**

The preoperative fasting serum fibrinogen levels and platelet counts of 58 patients with GBC were measured by AUV2700 automatic biochemical analyzer, as well as 60 patients with cholesterol polyps and 60 healthy volunteers. Kaplan–Meier survival analysis was applied to show the correction between fibrinogen levels and outcome after surgery.

**Results:**

The fibrinogen levels of patients with GBC were significantly higher than healthy gallbladder and cholesterol polyp of gallbladder (p < 0.001 and p < 0.001, respectively). In GBC, fibrinogen levels were associated with tumor depth (p = 0.001), lymph node metastasis (p = 0.002), distant metastasis (p < 0.001) and Tumor Node Metastasis (TNM) stage (p < 0.001). The levels in TNM stage IV disease were significantly higher than stage III or stage I + II disease (p = 0.048 and p < 0.001, respectively), and in TNM stage III disease were significantly higher than stage I + II disease (p = 0.002). Furthermore, the overall survival was better in low fibrinogen level group than in high fibrinogen level group (p < 0.001). However, thrombocytosis was not significantly associated with overall survivals (p > 0.05) in multivariate analysis.

**Conclusions:**

The preoperative serum fibrinogen levels and platelet counts might be reliable biomarkers for the occurance of disease, tumor depth, lymph node metastasis, distant metastasis and advanced TNM stage in patients with GBC. The serum fibrinogen levels might be a prognostic factor to predict outcome for GBC patients suffering from surgery treatment. Anticoagulation therapy might be considered to control cancer progression in future studies.

## Background

Primary gallbladder carcinoma (GBC) was one of the most common and malignant carcinoma of the biliary tract and was the sixth most common cancer of gastrointestinal tract [1]. Radical resection was the most effective treatment [2]. However, GBC was asymptomatic and was difficult to diagnose without suspicion in the early stage of occurrence, when the accurate diagnosis was made, radical cure often couldn’t be performed due to direct invasion into adjacent structures such as the hepatic artery or the portal vein [3], as well as metastasis via the lymphatic, perineural, and hematogenous routes [4], which resulting in poor overall prognosis [5]. Tumor depth,lymph node metastasis, distant metastasis and TNM stage were closely related to prognosis of GBC. So it was important to find effective biomarkers to recognize unique biological characteristics of GBC patients, and to guide more individualized treatment.

It is suggested that the alteration of coagulation pathways was related with tumor progression and poor prognosis in various malignancies [6, 7]. Hemostatic parameters were found abnormal in more than half of patients with metastatic disease [8]. Fibrinogen was an dimeric glycoprotein synthesized by hepatocytes [9], lung [10] and intestinal epithelium [11], which played a key role in blood clotting, fibrinolysis, inflammatory response, wound healing and neoplasia [12]. Under pathological states, the coagulant molecules from tumor cells which promoted the increase of fibrinogen [13]. Elevated serum fibrinogen levels reflected a thrombophilic state and influenced cancer cell growth, progression and metastasis. Recently, the serum fibrinogen levels were found to correlate with clinicopathological factors and prognosis in colorectal cancer [14], hepatic cancer [15], osteosarcoma [6, 7], malignant soft tissue tumors [11], pancreatic cancers [16], gastric cancer [17], and ovarian cancer [18]. These previous study suggested that the alteration of serum pre-therapeutic fibrinogen levels was a useful predictor of therapeutic response and prognosis. However, no study has determined the significance of serum fibrinogen levels in GBC patients as a predictor of survival after surgical treatment.

Platelet is another crucial player of the coagulation system. Platelet could facilitate metastasis by promoting disseminated tumor cell survival in the circulatory system, and extravasation and angiogenesis in the microenvironment of target sites [19]. Several previous studies have suggested that an elevated platelet count related to poor prognosis in solid malignancies, including colorectal cancer [14], hepatic cancer [15], osteosarcoma [6, 7], malignant soft tissue tumors [11], pancreatic cancers [16], gastric cancer [17], and ovarian cancer [18], etc.

In the present study, we examined the clinical significance of preoperative serum fibrinogen levels and platelet counts in GBC treated by surgical treatment.

## Methods

### Study population

A total of 58 patients who underwent surgery for GBC in Affiliated Union Hospital of Fujian Medical University between October 2008 and October 2017 were included in the study. Patients were already excluded because of incomplete clinical data, palliative radiotherapy or chemotherapy, preoperative mortality and lost to follow-up. Based on the clinical records, the following data were collected for each patient: age, gender, T stage and other miscellaneous characteristics. All cases were staged clinically according to the American Joint Committee on Cancer (AJCC, 7th). The clinic pathological characteristics of GBC patients were presented in Table 1.

Moreover, we choosed 60 patients as another group: 60 patients with cholesterol polyps, treated by surgery in the Affiliated Union Hospital of Fujian Medical University. The control group included 60 healthy volunteers. All cases didn’t have received any preoperative radiochemotherapy or transfusion, and all cases without coronary heart disease, cerebrovascular disease, arteriosclerosis, liver disease, COPD, thrombotic diseases, acute inflammation etc. All subjects provided their written informed consent, and the study was conducted with the approval of the institutional Ethics Committees of Fujian Medical University.

### Fibrinogen levels and platelet counts measurement

Venous blood samples were collected according to standard hospital clinical routine within 24 h to 1 week before surgery or anticancer after overnight fasting and measured by AUV2700 automatic biochemical analyzer (Olympus, Japan). Plasma fibrinogen levels and platelet counts greater than 4.0 g/L and 300 × 10^9^/L were defined as hyperfibrinogenemia and thrombocytosis, respectively, according to the normal reference range in our hospital.

### Follow-up assessments

All of the patients were followed by telephone interviews. The duration of follow-up was defined from the date of operation to the date of the last follow-up before the data were analyzed, or the date of death. The patients received follow-up until October 2020. The patients were followed up every 3 months during the first two postoperative years, every 6 months for the next year. Physical examination, chest radiography, peripheral blood tumor marker measurements (CA199 and AFP), and abdominal computed tomography or magnetic resonance imaging were performed during the follow-up period. The median follow-up duration was 13.6 months (range 1.5–36 months). The follow-up rate of this study was 87.9%.

### Comorbidity

Hypertension was delimited by using antihypertensive medications or in the presence of a diastolic BP of more than 90 mm Hg, a systolic BP level of more than 140 mm Hg. Diabetes mellitus was defined as the use of insulin or the use of oral hypoglycemic drugs or hemoglobin A1c (HbA1c) at a level higher than 6.5%. Ever-smokers, chewers were assumed for those who smoked, chewed at least once a day for a minimum period of 6 months. Former smokers were assumed for those who had stopped smoking at least 2 years before the interview. For the calculation of pack-years, the amount of tobacco in grams was estimated as 1 per cigarette, 0.5 per bidi and 2 per cigar, cheroot and chutta .

### Statistical analysis

The Shapiro-Wilk test was used to examine normal distribution of the fibrinogen level and platelet count data (p = 0.85 and p = 0.73,respectively). Because the fibrinogen level data and platelet counts showed normal distribution, all the data were presented by mean ± SD. The differences between these data and clinic pathological characteristics were evaluated using the Student t test for 2 groups and one-way analysis of variance (ANOVA) for more than 2 groups. Univariate Cox regression analyses were performed for baseline variables to determine indicators with poor prognosis and the multivariate analyses were carried out for the indicators with positive univariate analysis. The Kaplan-Meier survival analysis was used to estimate overall survival and the Mantel’s long-rank test was used to compare the differences in it. Pearson’s χ2 test was used to determine the significance of the differences for the dichotomous variables. Above statistical analyses were relied on standard SPSS (version 20, IBM, USA) and the Graphpad Prism 5 program. And all p values were based on a two-tailed statistical analysis. All the results were considered statistically significant when P < 0.05.

## Results

### Patient characteristics

**Fig. 1:**
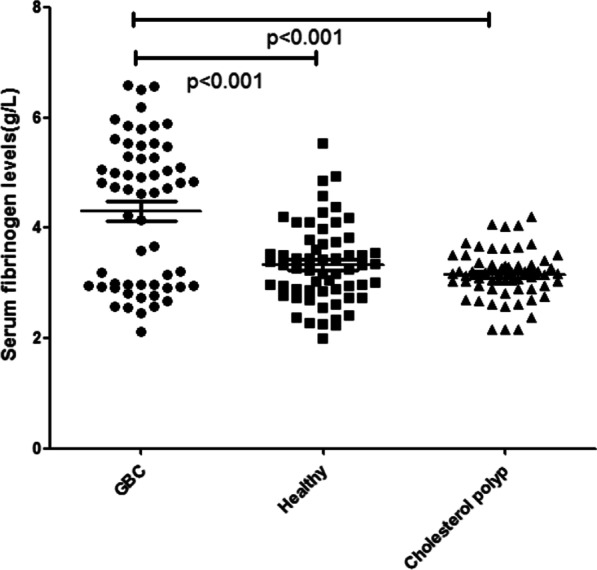
Difference between serum fibrinogen levels of GBC (58) and healthy volunteers (60), cholesterol polyp (60)

**Fig. 2:**
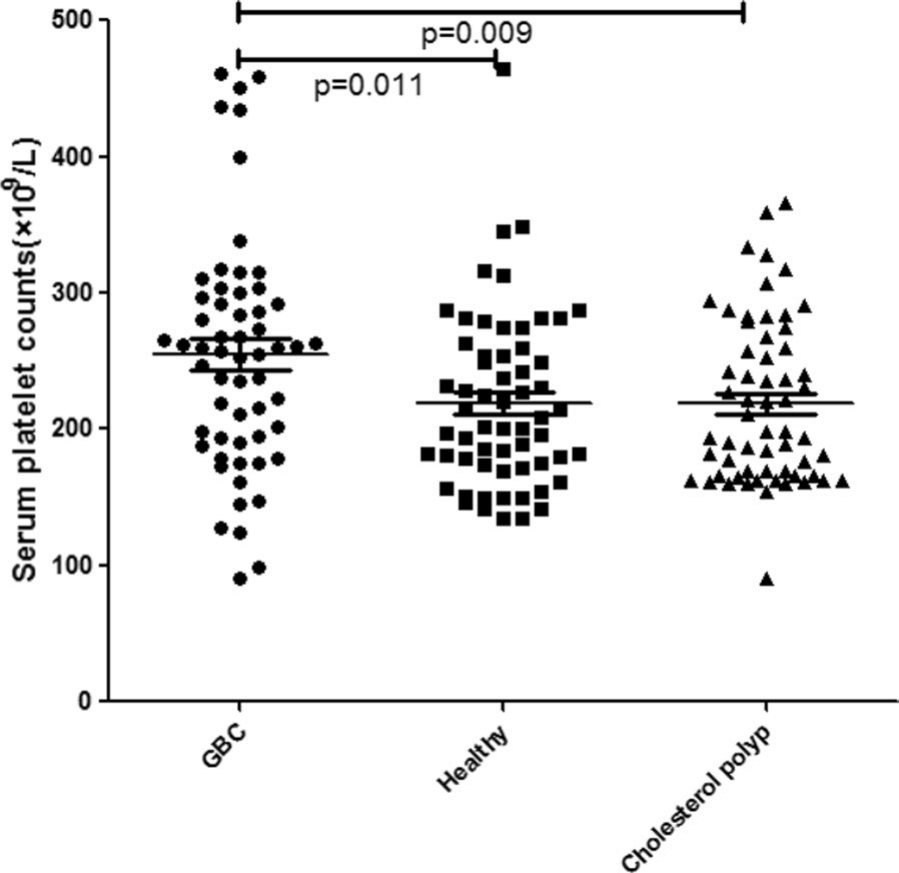
Difference between serum platelet counts of GBC (58) and healthy volunteers (60), cholesterol polyp (60)

Among 58 GBC patients of median age 63 years (range from 32 to 86 years), 33 (56.90%) were males and 25 (43.10%) were females, 42 (72.41%) were adenocarcinoma, 4 (6.90%) papillary carcinoma, 3 (5.17%) mucinous adenocarcinoma, 3 (5.17%) tubular adenocarcinoma, 2 (3.45%) squamous carcinoma, 1 (1.72%) was malignant adenofibroma, 1 (1.72%) sarcoma, 1 (1.72%) neuroendocrine tumors, 1 (1.72%) adenosquamous carcinoma, 31 (53.45%) were poorly differentiated carcinomas, 18(31.03%) moderately differentiated, and 9 (15.52%) well differentiated. The tumor location: 33 (56.90%) were in the neck, 13 (22.41%) in the body, 12 (20.69%) in the bottom. The tumor depth (T factor) : 1 (1.72%) Tis, 6 (10.34%) T2, 28 (48.28%)T3, 23 (39.66%)T4, 34(58.62%) patients were found with lymph node metastasis, and 18 (31.03%) were found with distant metastasis. All cases were staged clinically according to the American Joint Committee on Cancer (AJCC, 7th) ^[24]^: Among 60 cholesterol polyps patients of median age 59 years (range from 31 to 87 years), 27 (45.00%) were males and 33 (55.00%) were females, 19 (31.67%) with hypertension, 3 (5.00%) with diabetes, and 12 (20.00%) smokers; and 60 healthy volunteers of median age 64 years (range from 41 to 84 years), 36 (60%) were males and 24 (40.00%) were females, 10 (16.67%) with hypertension, 3 (5.00%) with diabetes, and 10 (16.67%) smokers. There was no significant difference in age, sex ratio and comorbidities between the three groups (p > 0. 05) (Table 1). The serum levels of fibrinogen and platelet counts in patients with GBC: In GBC patients, we found that the serum fibrinogen levels and platelet counts were significantly higher compared with healthy group (4.29 ± 1.33 vs. 3.25 ± 0.78 g/L, p < 0.001 and 254.90 ± 85.94 vs. 219.22 ± 63.13 × 10^9^/L, p = 0.011, respectively), with cholesterol polyp of gallbladder group (4.29 ± 1.33 vs. 3.16 ± 0.44 g/L, p < 0.001 and 254.90 ± 85.94 vs. 218.60 ± 60.05 × 10^9^/L, p = 0.007, respectively) (Figs. 1 and 2).

**Table.1:** Association between serum fibrinogen levels and platelet counts with clinicopathological characteristics

Item	Number	Fibrinogen (g/L)	P	Platelet count (×10^9^/L)	P
*Age (years)*					
< 65	17 (29.31)	4.33 ± 1.23		270.59 ± 98.90	
≥ 65	41 (70.69)	4.29 ± 1.39	0.893	248.39 ± 80.39	0.375
*Gender*					
Female	25 (43.10)	4.20 ± 1.17		246.40 ± 80.93	
Male	33 (56.90)	4.41 ± 1.54	0.557	261.33 ± 90.24	0.517
*Tumor location*					
Neck	33 (56.90)	4.50 ± 1.19		261.73 ± 81.45	
Others	25 (43.10)	4.01 ± 1.48	0.164	245.88 ± 92.44	0.492
*Tumor size*					
< 4 cm	40 (68.97)	3.29 ± 1.35		265.53 ± 78.19	
≥ 4 cm	18 (31.03)	3.35 ± 1.34	0.886	250.03 ± 91.17	0.529
*Histology*					
Adeno	42 (72.41)	3.87 ± 1.06		256.80 ± 87.51	
Other types	16 (27.59)	4.32 ± 1.35	0.519	229.25 ± 63.99	0.541
*Histological grading*					
Poorly	31 (53.45)	4.59 ± 1.34		262.48 ± 81.01	
Moderately	18 (31.03)	4.00 ± 1.18		244.00 ± 107.75	
High	9 (15.52)	3.83 ± 1.47	0.174	250.56 ± 53.14	0.764
*Tumor depth*					
0–II	7 (12.07)	2.74 ± 0.69		247.71 ± 35.99	
III–IV	51 (87.93)	4.50 ± 1.26	0.001	255.88 ± 90.86	0.816
*LN metastasis*					
N0	24 (41.38)	1.87 ± 0.58		249.47 ± 78.78	
N1	16 (27.59)	3.12 ± 0.88		251.45 ± 93.00	
N2	18 (31.03)	4.41 ± 0.90	0.002	262.57 ± 88.13	0.879
*Distant metastasis*					
M0	40 (68.97)	2.69 ± 1.13		239.85 ± 84.39	
M1	18 (31.03)	4.62 ± 0.69	0.0002	288.33 ± 81.90	0.046
*TNM stage*					
I+II	7 (12.07)	2.69 ± 0.62		247.86 ± 36.04	
III	25 (43.11)	4.16 ± 1.12		231.96 ± 92.87	
IV	26 (44.82)	4.85 ± 1.30	0.0002	278.85 ± 84.32	0.146
*Liver invasion*					
Present	28 (48.28)	4.64 ± 1.24		274.86 ± 92.28	
Absent	30 (51.72)	3.97 ± 1.35	0.055	236.27 ± 69.13	0.088
*Choledoch invasion*					
Present	21 (36.20)	4.72 ± 1.38		280.86 ± 98.48	
Absent	37 (63.80)	4.05 ± 1.26	0.066	240.16 ± 75.43	0.083
*Diabetes*					
Present	9 (15.52)	4.39 ± 1.43		231.11 ± 47.61	
Absent	49 (84.48)	4.27 ± 1.33	0.799	259.27 ± 90.92	0.371
*Hypertension*					
Present	15 (25.86)	4.83 ± 1.44		265.53 ± 69.98	
Absent	43 (74.14)	4.10 ± 1.26	0.069	251.19 ± 91.30	0.582
*Smoking*					
Present	14 (24.14)	4.05 ± 1.27		247.07 ± 90.41	
Absent	44 (75.86)	4.37 ± 1.36	0.441	257.39 ± 85.39	0.699

### Correlation between plasma fibrinogen levels, platelet counts, and clinicopathological parameters in patients with GBC

The serum fibrinogen levels in preoperative GBC ranged from 1.46 to 6.60 g/L (4.29 ± 1.33 g/L). The occurrence of hyperfibrinogenemia was 58.62% (34/58, cut-off value 4.0 g/L). Hyperfibrinogenemia was found to be positively correlated with a increased depth of invasion (p = 0.001), LN metastasis (p = 0.002), distant metastasis (p < 0.001) and advanced pathological stages (p < 0.001). No significant correlation was identified between fibrinogen levels and tumor size, tumor location, histological type and histological grading (Table 1). The platelet counts ranged from 91 × 10^9^/L to 461 × 10^9^/L (254.90 ± 85.94 × 10^9^/L). The occurrence of thrombocytosis was 24.14% (14/58, cut-off value 300 × 10^9^/L). Thrombocytosis was positively correlated with distant metastasis (p = 0.046). There was no significant correlation between the platelet count and the larger tumor size, tumor location, increased depth of invasion, LN metastasis and advanced pathological stages (Table 1).

### Analysis of the prognostic factors

To further explore the risk predictors of poor prognosis, the univariate and multivariate analyses of poor prognosis data which were presented in Table 2. The univariate analyses showed that age (P = 0.031), LN metastasis (P < 0.001), TNM staging (P = 0.001), platelet counts (P = 0.046), liver invasion (P = 0.004), choledoch invasion (P = 0.002), and fibrinogen levels (P = 0.008) were predictive factors for poor prognosis. Subsequently, multivariate analyses indicated that LN metastasis(OR: 4.301; 95% Cl 1.612–11.483; P = 0.002), TNM staging (OR: 1.003; 95% Cl 0.386–2.806; P < 0.001), liver invasion (OR: 2.231; 95% Cl 1.172–4.875; P = 0.023), choledoch invasion (OR: 2.765; 95% Cl 1.287–5.769; P < 0.001), and fibrinogen levels (OR: 1.012; 95% Cl 0.682–1.876; P = 0.034) were predictive factors for poor prognosis. Regarding prognosis, the patient in group with low LN metastasis, TNM staging, liver invasion and choledoch invasion was superior than the higher group. The four predictive factors we have detected can actually represent the tumor stage, invasion and metastasis degree, which have been used as conventional prognostic risk factors in clinical practice. In addition, we found that the serum fibrinogen level (≥ 4 g/L) was also prognostic risk factor for poor prognosis. Hence, survival analyse was performed for high-risk and low-risk groups based on this prognostic risk factor.

**Table.2:** Uni-and multivariate analyses of factors predicting overall survival

Variable	Univariate analysis			Multivariate analysis		
	OR	95 %CI	P	OR	95 %CI	P
Age	2.189	1.109–4.323	**0.031**	1.785	0.983–4.843	0.12
Gender	0.572	0.298–1.095	0.092			
Tumor depth	1.37	0.701–2.695	0.354			
LN metastasis	4.412	2.128–9.148	**< 0.001**	4.301	1.612–11.483	**0.002**
TNM staging	1.88	1.442–2.451	**0.001**	1.003	0.386–2.806	**< 0.001**
Surgery method	1.09	0.552–2.132	0.809			
Differentiation degree	0.647	0.408–1.025	0.064	1.247	0.752–1.793	0.127
Platelet counts	0.971	0.826–1.279	**0.046**			
Pathology	0.959	0.339–2.708	0.937			
Jaundice	1.529	0.773–3.026	0.222			
Cholelithiasis	1.501	0.790–2.850	0.215			
Tumour location	1.623	0.931–2.331	0.321			
Liver Invasion	2.648	1.370–5.117	**0.004**	2.321	1.172–4.875	**0.023**
Choledoch Invasion	3.053	1.498–6.224	**0.002**	2.765	1.287–5.769	**0.001**
Diabetes	0.887	0.347–2.269	0.802			
Hypertension	0.92	0.448–1.889	0.82			
Smoking	2.02	1.493–3.111	0.112			
Fibrinogenlevels	1.493	0.792–1.787	**0.008**	1.012	0.682–1.876	**< 0.001**

### Correlation between serum fibrinogen levels and platelet counts in the patients with GBC

**Fig. 3:**
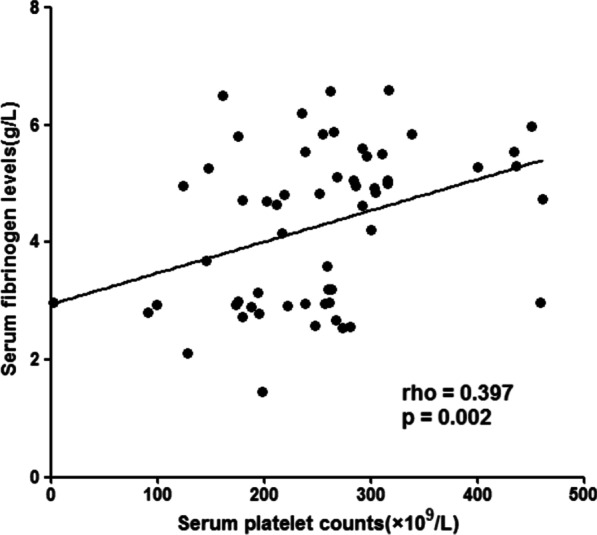
Correlation between serum fibrinogen levels and platelet counts in GBC

To evaluate the correlation between serum fibrinogen levels and platelet counts, we analyzed it with the Spearman Rank Correlation. A significant positive correlation was found between serum fibrinogen levels and platelet counts (r = 0.397, p = 0.002) (Fig. 3).

## Correlation between serum fibrinogen levels/platelet counts and prognosis

**Fig. 4:**
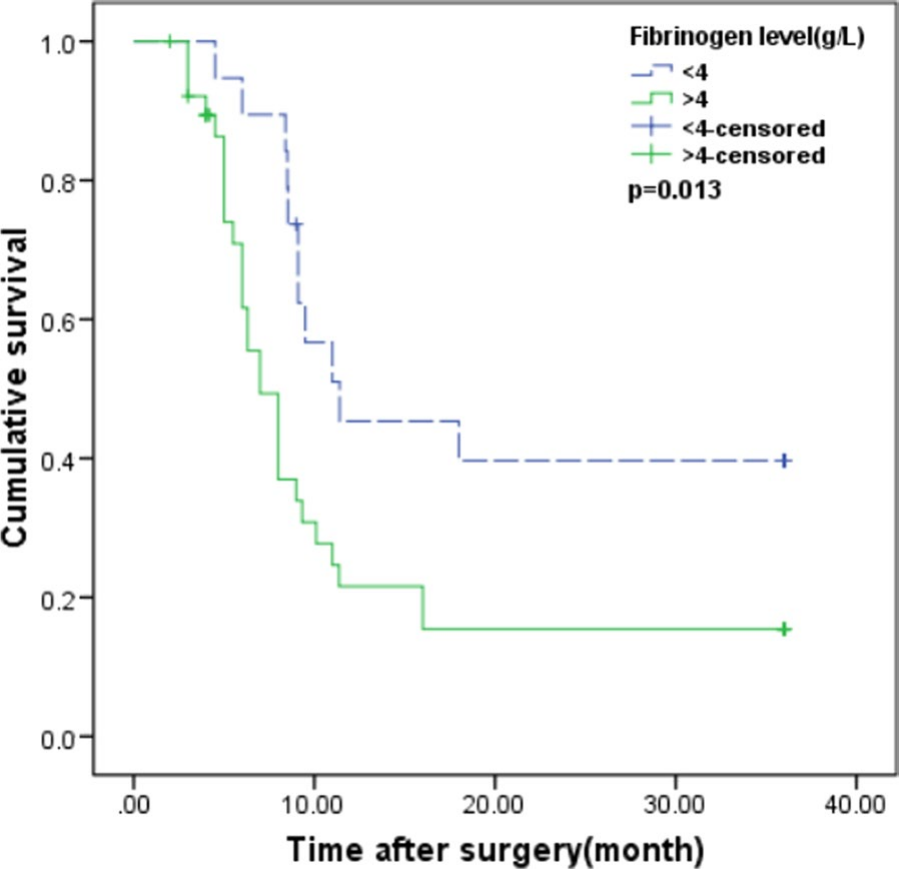
Survival curve according to serum fibrinogen levels for patients

In our study, the median survival time was 8.2 months, and the median follow-up time was 13.6 months (range from 1.5 to 36 months). The follow-up rate reached 87.9%. The 3-year overall survival rate was 20.7%.The mean survival time of patients with high serum fibrinogen level (≥ 4 g/L) was 7.0 months (n = 39, 95% confidence interval (CI): 8.04–15.43),while the low serum fibrinogen level (< 4 g/L) was 11.4 months (n = 19, 95% confidence interval (CI): 13.95–26.11) (p = 0.001). The overall survival was worse in high fibrinogen levels group than in low fibrinogen levels group (p < 0.001) (Fig. 4). Furthermore, the levels in TNM stage IV disease were significantly higher than stage III or stage I+II disease (p = 0.048 and p < 0.001, respectively),and in TNM stage III disease were significantly higher than stage I+II disease (p = 0.002). However, platelet counts were not associated with overall survival (p > 0.05), but were significantly higher (300 × 10^9^/L) in GBC with distant metastasis disease (p = 0.046).

## Discussion

Fibrinogen is a soluble protein synthesized by hepatocytes [9], lung [10] and intestinal epithelium [11], which played a key role in blood clotting, fibrinolysis, inflammatory response, wound healing and neoplasia [12]. Considerable attention has been paid to the association between the progression of malignancies and hyperfibrinogenemia/thrombocytosis. Elevated serum fibrinogen levels and platelet counts were found in various malignancies,such as colorectal cancer [14], hepatic cancer [15], osteosarcoma [6, 7], malignant soft tissue tumors [11], pancreatic cancers [16], gastric cancer [17], and ovarian cancer [18], etc. Our study showed that the incidence of hyperfibrinogenemia and thrombocytosis were 67.2% (39/58, cut-off value 4.0 g/L) and 24.1% (14/58, cut-off value 300 × 109/L), respectively, in the GBC patients. And the activation of fibrinolysis and coagulation in GBC patients was frequently related to increased depth of invasion, advanced pathological stages and eventual worse outcomes, so hypercoagulability might be served as a sign of a more aggressive disease. However, we found that hyperfibrinogenemia was associated with poor overall survivals in univariate analysis, but not an independent predictor for prognosis in multivariate analysis. Therefore, the serum fibrinogen might serve as not only a local regulator but also a systematic mediator. We also found that thrombocytosis was not associated with depth of invasion, lymph node metastasis, pathological stages, or overall survival, but was significantly higher in GBC with distant metastasis disease. We also observe a significant correlation serum fibrinogen levels and platelet counts (r = 0.397, p = 0.002).

The previous study demonstrated that increased expression of thrombomodulin might reduce the migration of cancer cells by restraining PI3K and Akt [20]. Furthermore,increasing evidence showed that the anticoagulant drugs, such as warfarin and heparin, have antitumor effects both in vivo and in vitro [21]. Anticoagulants, especially the low molecular heparin, have a splendid antitumor effect without fatal bleeding and venous thromboembolism [22]. Other factors including hepatic free fatty acids, non-enzymatic oxysterols or diabetes, can facilitate the progression of other liver diseases including fatty liver, HCC and etc. [23–25].

And for all we knew, no research was carried out to analyzed the clinical significance of serum fibrinogen levels in GBC patients. In the study, we firstly analyzed the clinical significance of pretreatment serum fibrinogen levels in 60 GBC treated by surgery treatment without neoadjuvant treatment or transfusion. Our study showed that hyperfibrinogenemia was significantly related to advanced disease.

The platelet counts have been reported as prognostic factors in various malignancies. Wang et al. proposed that platelet counts were closely correlated with GBC prognosis and could be used to identify the population with a poorer prognosis after surgery [26]. Feng et al. found that preoperative platelet count was a predictive factor for long-term survival in ESCC, especially in node-positive patients [27]. Ohuchi M et al. found that a high platelet count is associated with tumor progression and poor survival in patients with esophageal cancer [28]. For thrombocytosis, even inflammation-based scores, such as PLR, was established to analyzes the prognosis of maglinancies [29–32]. The reason for our finding conflicted with previous studies may be that our sample size was relatively too little and the patients with relative advanced stage compared with previous studies.

Although large numbers of studies have shown that the coagulation pathways were associated with tumor progression, the exact mechanism remains inclusive. Fibrinogen was an important component of the extracellular matrix, and hold matrix, tumor cells together through integrins or intercellular adhesion molecule [33]. Fibrinogen generated proliferative signals by binding growth factors, such as fibroblast growth factors (FGF)-2 [34] and vascular endothelial cell growth factors (VEGF) [35], which regulated cellular proliferation and angiogenesis. Fibrinogen deposition around tumor cells enhances the interaction between these cells and platelets, thereby forming microemboli in target organs [36]. Fibrinogen layers help tumor cells block natural killer cytotoxicity with thrombin, which might protect tumor cells from the host’s immune system [37].

Platelets, which were produced by mature bone marrow megakaryocytes, and involved in the physiological process of coagulation and in the growth and metastasis of tumors by adhering to, aggregating and releasing their angiogenic contents [38, 39]. Platelets might release various chemokines, cytokines, proteases, and procoagulants [40]. These agents played key roles in arresting hemorrhage after tissue trauma or vascular injury,blood clotting, tumor cell growth and angiogenesis [41]. Platelets enhance tumor metastasis by expressing immunoregulatory proteins, such as the glucocorticoid induced TNF-related protein to protect tumor cells from the innate immune system [42, 43]. Furthermore, A recent study demonstrated that the interactions between platelets and tumor cells facilitated metastasis by promoting epithelial mesenchymal transition through the TGFB/SMAD and NFKB pathways.They also found that inhibition of these two pathways solely could suppress metastasis in vivo [44].

There are several limitations to our study. First, our cohort was retrospective, and the sample size is relatively small. In order to select patients with a more uniform background, we only included GBC patients treated by potential curative surgery and excluded those received neoadjuvant treatment, which may also limit the general application of the results. Furthermore, larger prospective studies will be needed to confirm these preliminary results.

## Conclusions

Our study demonstrated that hyperfibrinogenemia was a valuable predictor for disease progression and prognosis in GBC. Anticoagulation therapy might be considered to control cancer progression in future studies. Additionally, the serum fibrinogen levels can be a feasible biomarker to assist clinicians in working out better individualization of their therapeutic approach based on the risk stratification.

## Data Availability

The data sets analyzed during the current study are available from the corresponding author on reasonable request.

## Abbreviations

GBC: Gallbladder carcinoma
TNM: Tumor Node Metastasis
AJCC: American Joint Committee on Cancer
ANOVA: One-way analysis of variance
CI: Confidence interval
FGF: Fibroblast growth factors
VEGF: Vascular endothelial cell growth factors
